# Sickle Cell Hepatic Vaso‐Occlusive Crisis: A Case Report

**DOI:** 10.1155/crh/7991112

**Published:** 2026-05-25

**Authors:** Bhimla Rambaran

**Affiliations:** ^1^ Department of Hematology and Oncology, San Fernando General Hospital, San Fernando, Trinidad and Tobago, health.gov.tt

## Abstract

Sickle cell disease (SCD) is an inherited hemoglobinopathy caused by a point mutation in the β‐globin gene, resulting in hemoglobin S polymerization under hypoxic conditions. This process leads to erythrocyte dehydration, impaired deformability, hemolysis, and recurrent vaso‐occlusion, which may culminate in acute painful crises and progressive organ damage. Hepatic involvement is a recognized but underreported complication of SCD and can range from mild biochemical abnormalities to life‐threatening hepatic vaso‐occlusive crisis (VOC). This case report presents a 25‐year‐old woman, with homozygous sickle cell anemia who developed a rapidly progressive hepatic VOC following a mild respiratory illness. Her clinical course was marked by acute abdominal pain, severe transaminitis, cholestasis, escalating transfusion requirements, and radiologic evidence of hepatomegaly. Clinical and biochemical improvement was observed following a multimodal management approach that included supportive care, corticosteroids, blood transfusion, and the addition of intravenous metronidazole. This case highlights hepatic VOC as a serious manifestation of SCD and explores the potential role of metronidazole as an adjunctive therapy, given its immunomodulatory and anti‐inflammatory properties. Further investigation is warranted to clarify its therapeutic role in sickle cell–related vaso‐occlusive complications.

## 1. Introduction

Sickle cell disease (SCD) is a hereditary hemoglobin disorder characterized by the presence of hemoglobin S (HbS), which predisposes red blood cells (RBCs) to polymerization and deformation under deoxygenated conditions [1]. These pathologic changes promote hemolysis, endothelial adhesion, and recurrent vaso‐occlusion, leading to acute painful crises and cumulative end‐organ injury [2]. The clinical severity can vary significantly, ranging from mild painful episodes to life‐threatening complications [1, 2].

Hepatic involvement in SCD, collectively termed sickle hepatopathy, encompasses a spectrum of clinical entities including acute sickle hepatic crisis, hepatic sequestration, intrahepatic cholestasis, and chronic liver disease. Acute sickle hepatic crisis occurs in approximately 10% of patients with sickle cell anemia [3]. It typically presents with right upper quadrant pain, hepatomegaly, jaundice, and marked elevations in aminotransferases. At the microvascular level, sickled erythrocytes obstruct hepatic sinusoids, resulting in ischemia, inflammation, and hepatocellular injury [4].

Current therapeutic strategies in SCD primarily target the prevention of HbS polymerization, which is central to the pathogenesis of hemolysis and vaso‐occlusion [5]. One approach involves targeting erythrocyte dehydration pathways, such as the Gardos channel which is a calcium‐activated potassium channel known to contribute to cell dehydration and sickling. Separately, increasing attention has been directed toward the role of systemic inflammation, leukocyte adhesion, and the gut microbiome in the pathogenesis of vaso‐occlusion [5].

Metronidazole, a nitroimidazole antimicrobial agent, has been shown to exert anti‐inflammatory and immunomodulatory effects beyond its antimicrobial activity [6]. Emerging evidence suggests that antibiotic‐mediated alterations in gut microbiota may influence neutrophil activation and adhesion, thereby modifying the risk and severity of vaso‐occlusive crises [7]. This case report explores the pathophysiology of hepatic vaso‐occlusion and describes the clinical course of a patient with severe hepatic involvement in whom intravenous metronidazole was used as part of a multimodal treatment strategy.

## 2. Case Presentation

A 25‐year‐old Afro‐Caribbean woman with known sickle cell disease (HbSS) presented to the emergency department with a three‐day history of fever and nonproductive cough. She was well known to the hematology service and had been diagnosed with SCD at the age of seven. Her past medical history was notable for a previous hospitalization 1 year prior for a painful vaso‐occlusive crisis (VOC). Her steady‐state hemoglobin was approximately 7‐8 g/dL. Prior to admission, she was taking folic acid routinely and uses acetaminophen as needed for intermittent pain.

On examination, she was hemodynamically stable and afebrile. Vital signs on admission included a pulse rate of 93 beats per minute, blood pressure of 137/87 mmHg, respiratory rate of 20 breaths per minute, oxygen saturation of 96% on room air, temperature of 37°C, and a random blood glucose level of 126 mg/dL. Cardiovascular examination was unremarkable. Respiratory examination revealed mildly reduced air entry at the lung bases without wheezes or crackles. Oxygen saturation was maintained on room air. Initial laboratory investigations demonstrated a hemoglobin level of 8.1 g/dL, consistent with her steady‐state baseline (7‐8 g/dL), and an elevated C‐reactive protein (CRP) of 21.7 mg/L. Other laboratory parameters were within normal limits.

The patient was admitted for inpatient management and treated with intravenous hydration, nebulized bronchodilator therapy consisting of Ventolin (5 mg) and Atrovent (500 mcg) administered every six hours, and oral prednisolone at a dose of 40 mg daily for five days. Empiric antimicrobial therapy with intravenous ceftriaxone was initiated once daily. Additional supportive measures included chest physiotherapy, strict monitoring of oxygen saturation, and general supportive care.

Over the subsequent 4 days, she demonstrated clinical improvement with resolution of respiratory findings and a downtrend in inflammatory markers.

On Day 5 of hospitalization, she acutely developed generalized weakness, dizziness, severe bilateral lower limb joint pain, and right upper quadrant abdominal pain. Vital signs revealed blood pressure of 178/98 mmHg, heart rate of 89 beats per minute, respiratory rate of 20 breaths per minute, and oxygen saturation of 95% on room air. Abdominal examination revealed new‐onset hepatomegaly, which had not been previously documented during the patient’s prior admissions or steady‐state clinic visits, and which was also not noted during the first few days of this admission.

Repeat laboratory investigations revealed marked hepatic dysfunction, with aspartate aminotransferase (AST) of 1110 U/L, alanine aminotransferase (ALT) of 392.4 U/L, alkaline phosphatase of 680 U/L, and gamma‐glutamyl transferase of 735 U/L. Total bilirubin was 6.09 mg/dL with a direct fraction of 5.52 mg/dL. Lactate dehydrogenase (LDH) was elevated at 2520 U/L. White blood cell count was 29.0 × 10^9^/L, platelet count was 405 × 10^9^/L, and hemoglobin was 8.2 g/dL. Renal function remained preserved. Serial laboratory trends are summarized in Table 1.

**TABLE 1:** Liver enzymes and inflammatory markers during hospitalization.

Test	27/11/2024	28/11/2024	29/11/2024	01/12/2024	02/12/2024	03/12/2024	04/12/2024	05/12/2024	06/12/2024	09/12/2024	10/12/2024	13/12/2024
GGT (U/L)	278	—	—	735	1051	672	636	466	868	615	570	414
AST (IU/L)	72.4	—	—	1110	908	263.9	136.7	53.8	232.9	29.3	31.9	44.9
ALT (IU/L)	110.6	—	—	392.4	461.2	224.2	170.3	100	119.8	48.4	43.8	33.3
ALKP (U/L)	448	—	—	680	984	1245	1228	839	1271	820	714	556
LDH (U/L)	—	—	—	2520	5093	5311	4945	3327	2799	1635	1494	1179
CRP (mg/dL)	21.7	—	—	4.43	11.7	41.4	40.2	—	34.3	10.4	5.1	—
WBC (× 10^3^/μL)	28.1	—	—	29	36	48	36	29.8	36	—	20	—

**FIGURE 1:**
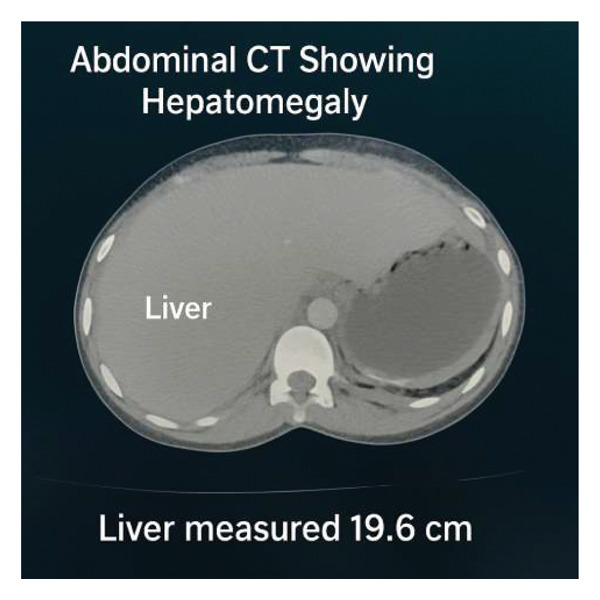
Abdominal computed tomography scan demonstrating hepatomegaly.

Computed tomography of the abdomen demonstrated hepatomegaly (Figure 1), cardiomegaly, and bilateral ovarian cysts. No focal hepatic lesions or biliary ductal dilatation was identified.

Over the subsequent 48 h, the patient experienced progressive clinical deterioration, accompanied by a marked decline in hemoglobin from 8.1 g/dL on admission to 5.8 g/dL on serial monitoring. In view of this acute anemia and overall clinical status, urgent RBC transfusion was administered, and admission to the intensive care unit was considered.

Given persistently deranged liver function tests, leukocytosis, and ongoing clinical decline, intravenous ceftriaxone was discontinued and intravenous metronidazole (500 mg three times daily) was initiated. The patient continued to receive intravenous fluids, supplemental oxygen via face mask for intermittent desaturation, and oral prednisolone at 40 mg daily.

Following transfusion of one unit of packed RBCs, hemoglobin improved to 7.5 g/dL. Over the subsequent 4 days, liver enzymes declined progressively, and the patient demonstrated significant clinical improvement, including resolution of abdominal pain, improved appetite, and stable oxygenation on room air. Prednisolone was subsequently tapered to 20 mg daily.

After 14 days of hospitalization, the patient self‐discharged against medical advice. Outpatient follow‐up was arranged. At the 3‐week follow‐up, liver function tests had normalized, and the patient resumed routine hematology clinic follow‐up.

## 3. Discussion

This case highlights the complex and rapidly evolving nature of SCD when hepatic VOC develops. Although hepatic involvement is a recognized manifestation of SCD, acute hepatic VOC remains relatively uncommon and may be easily overlooked in the setting of concurrent infection or systemic illness. In this patient, a mild respiratory infection preceded abrupt hepatic dysfunction, suggesting that systemic inflammation and hypoxia may have acted as precipitating factors for intrahepatic vaso‐occlusion [1–4].

### 3.1. Hepatic Vaso‐Occlusion in SCD

SCD results from a mutation in the β‐globin gene, producing HbS, which polymerizes under deoxygenated conditions [2]. This polymerization causes the RBCs to become rigid, dehydrated, and sickle shaped, impairing their ability to traverse the microvasculature and increasing their adhesiveness to endothelial cells [2]. Within the liver, these altered RBCs become trapped in the sinusoidal microcirculation, leading to impaired perfusion, hepatocellular ischemia, and subsequent inflammatory injury [4]. Clinically, this process manifests as right upper quadrant pain, hepatomegaly, jaundice, and marked elevations in aminotransferases and LDH, all of which were observed in this case [4]. Several mechanisms contribute to this cascade. Dehydration, hypoxia, acidosis, and systemic inflammation can each promote erythrocyte sickling and endothelial activation [5].

The abnormal calcium handling in sickle RBCs plays a central role; elevated intracellular calcium levels activate the Gardos channel, leading to potassium and water efflux, furthering RBC dehydration and sickling [5]. The resulting cell rigidity increases the risk of mechanical trapping in the hepatic sinusoids, leading to ischemia and hepatocellular injury, as reflected in the dramatic rise in ALT and LDH in this patient [8].

### 3.2. Risk Factors and Clinical Predictors

Hepatic VOC in SCD is often precipitated by a combination of patient related and environmental factors. Reported risk factors include severe genotypes such as homozygous HbSS, dehydration, infection, hypoxia, and prior vaso‐occlusive episodes. In addition, underlying hepatic vulnerability, including iron overload from repeated transfusions and pre‐existing liver dysfunction, may increase susceptibility to hepatic involvement. In this case, the preceding respiratory illness likely contributed to systemic inflammation and hypoxia, acting as a trigger for intrahepatic vaso‐occlusion.

Clinical predictors of disease severity and poor outcomes include markedly elevated bilirubin levels, particularly above 20 mg/dL, significant transaminitis, coagulopathy, renal impairment, and the presence of encephalopathy. Severe forms such as sickle cell intrahepatic cholestasis are associated with high morbidity and mortality, underscoring the importance of early recognition and aggressive management.

### 3.3. Inflammation, Leukocyte Activation, and the Microbiome

Emerging research has implicated activated neutrophils and systemic inflammation in the pathogenesis of VOC. Neutrophils contribute to vaso‐occlusion via endothelial adhesion, cytokine release, and generation of reactive oxygen species (ROS) which interact with sickled erythrocytes and contribute to microvascular obstruction [7].

Recent experimental data have also highlighted the influence of the gut microbiota on neutrophil aging, activation, and adhesion. Alterations in microbial composition have been shown to modulate systemic inflammatory tone and vaso‐occlusive risk in animal models of SCD. These observations raise the possibility that therapies capable of modifying gut microbial populations or dampening inflammation may indirectly influence the severity of VOC [9].

### 3.4. Potential Role of Metronidazole

To date, there is no published evidence supporting the use of metronidazole specifically in hepatic VOC. Metronidazole is conventionally used for the treatment of anaerobic and protozoal infections [10] and is widely utilized in hepatic infections, particularly in the treatment of anaerobic and amoebic liver abscesses, where it demonstrates excellent hepatic tissue penetration and antimicrobial efficacy.

It also possesses anti‐inflammatory and immunomodulatory properties that may be relevant in SCD. In hypoxic environments, metronidazole undergoes reductive activation, generating intermediates that can attenuate oxidative stress. Given the contribution of oxidative injury to sickle‐related tissue damage, this mechanism may have therapeutic relevance during VOC.

In addition, metronidazole has been shown to suppress nuclear factor kappa B (NF‐κB) signaling and reduce the production of pro‐inflammatory cytokines and adhesion molecules [6]. By downregulating these pathways, metronidazole may reduce endothelial activation and neutrophil adhesion critical events in VOC [10].

An additional hypothesis relates to metronidazole’s imidazole structure, which it shares with other agents such as clotrimazole that have demonstrated the inhibition of the Gardos channel in vitro. While metronidazole has not been formally evaluated as a Gardos channel inhibitor, its structural similarity raises the possibility of overlapping effects on erythrocyte hydration and sickling [12, 13]. This potential mechanism remains speculative but warrants further investigation.

### 3.5. Proposed Risk Stratification

Based on available literature and clinical observations, a pragmatic risk stratification approach to hepatic VOC may be considered. Mild disease may present with isolated hyperbilirubinemia and minimal enzyme elevation without systemic compromise. Moderate disease is characterized by symptomatic hepatic involvement with rising transaminases and bilirubin. Severe disease includes extreme hyperbilirubinemia, coagulopathy, encephalopathy, or multiorgan dysfunction. This classification may assist in guiding clinical decision‐making, including the need for escalation of care such as exchange transfusion and intensive monitoring.

### 3.6. Multimodal Management and Clinical Implications

The patient’s clinical improvement occurred in the context of a comprehensive management strategy targeting multiple aspects of VOC pathophysiology. Aggressive intravenous hydration likely improved microvascular flow and blood viscosity, while supplemental oxygen reduced further HbS polymerization. Blood transfusion corrected acute anemia and improved oxygen delivery. Corticosteroids may have contributed to attenuation of systemic inflammation, although their role in VOC remains controversial [11].

The temporal association between the initiation of intravenous metronidazole and subsequent clinical and biochemical improvement is notable. However, causality cannot be established from a single case, and the observed recovery likely reflects the combined effects of supportive care and transfusion therapy. Nonetheless, this case raises the possibility that metronidazole may serve as a useful adjunctive therapy in selected patients with severe VOC, particularly in settings where treatment options are limited [10].

Hepatic VOC may have long‐term implications for liver function, particularly with recurrent episodes, potentially contributing to chronic cholestasis, fibrosis, and, rarely, cirrhosis. Imaging findings are often nonspecific but commonly include hepatomegaly and features of sinusoidal congestion in the absence of biliary obstruction, emphasizing the importance of clinical and biochemical correlation [14]. Accordingly, patients require longitudinal follow‐up with serial liver function monitoring and hematology review to reduce recurrence risk and optimize disease‐modifying therapy. On continued outpatient follow‐up, the patient described in this report has remained clinically asymptomatic, with sustained normalization of liver function tests.

## 4. Limitations and Future Directions

This report is limited by its single‐patient design, the absence of photo documentation such as a peripheral blood film, and the lack of mechanistic data directly linking metronidazole to reduced sickling or inflammation. Viral hepatitis serologies were not performed in this case, which represents an additional limitation; however, the clinical context, biochemical pattern, and subsequent clinical course supported a diagnosis of hepatic VOC. Further studies are needed to clarify whether metronidazole exerts clinically meaningful effects on erythrocyte hydration, leukocyte activation, or microbiota‐mediated inflammation in SCD [5]. Prospective studies and randomized trials would be required to determine its safety, efficacy, and optimal role in the management of vaso‐occlusive complications.

## 5. Conclusion

Hepatic VOC is an uncommon but potentially severe complication of SCD, characterized by hepatic ischemia, elevated liver enzymes, and clinical deterioration [14, 15]. Prompt recognition and comprehensive supportive care are critical to prevent irreversible liver damage. This case raises the possibility that metronidazole may have a role as an adjunctive agent in the management of hepatic VOC, potentially related to its anti‐inflammatory, antioxidant, and immunomodulatory properties in hypoxic tissues [10]. While this observation is preliminary, it underscores the need for further research to elucidate metronidazole’s mechanisms of action and to evaluate its efficacy in larger cohorts. Expanding the pharmacologic options for VOC may ultimately improve outcomes and quality of life for patients with SCD.

## Author Contributions

Bhimla Rambaran was involved in the clinical care of the patient, conception and design of the case report, data acquisition and interpretation, manuscript drafting, critical revision, and final approval of the version to be published.

## Funding

The author received no specific funding for this work.

## Ethics Statement

Ethical approval was not required for this case report in accordance with local institutional policy, as it describes a single patient with no identifiable information. The report was prepared in accordance with institutional ethical standards.

## Consent

Written informed consent was obtained from the patient for publication of this case report and any accompanying images. A copy of the written consent is available for review by the Editor‐in‐Chief of the journal upon reasonable request.

## Conflicts of Interest

The author declares no conflicts of interest.

## Supporting Information

Additional supporting information can be found online in the Supporting Information section.

## Supporting information


**Supporting Information** CARE reporting guideline statement: This case report was prepared in accordance with the CARE (CAse REport) reporting guidelines. A completed CARE checklist is provided as a supporting file.

## Data Availability

All data relevant to this case report are included within the article. Additional details are available from the corresponding author upon reasonable request.
