# Supplementary material for: Sickle Cell Hepatic Vaso‐Occlusive Crisis: A Case Report
Source: Case Rep Hematol. 2026 May 25;2026:7991112. doi: 10.1155/crh/7991112 (PMC13201256; doi:10.1155/crh/7991112)
Supplement: Supplementary file 1 — Supporting Information CARE reporting guideline statement: This case report was prepared in accordance with the CARE (CAse REport) reporting guidelines. A completed CARE checklist is provided as a supporting file. [file CRH-2026-7991112-s001.docx]

# CARE Reporting Checklist for Sickle Cell Hepatic Vaso-Occlusive Crisis: A Case Report

| CARE Item | Description / Status |
| --- | --- |
| Title | The title includes the words 'case report' and identifies the key clinical issue. |
| Keywords | Relevant keywords including 'sickle cell disease', 'hepatic vaso-occlusive crisis', and 'metronidazole' are provided. |
| Abstract | The abstract summarizes the background, case presentation, intervention, and clinical outcome. |
| Introduction | The introduction outlines the clinical relevance and rarity of hepatic vaso-occlusive crisis. |
| Patient Information | Patient demographics, diagnosis (HbSS), and relevant medical history are described. |
| Clinical Findings | Presenting symptoms, physical examination findings, and vital signs are reported. |
| Timeline | The clinical course is described chronologically within the case presentation. |
| Diagnostic Assessment | Laboratory findings and imaging results are detailed; limitations of investigations are acknowledged. |
| Therapeutic Intervention | Supportive care, transfusion, antibiotics, corticosteroids, and metronidazole therapy are described. |
| Follow-up and Outcomes | Clinical improvement and normalization of liver function tests on follow-up are reported. |
| Discussion | The discussion addresses diagnosis, management, implications, and limitations. |
| Patient Perspective | Not available; patient perspective was not obtained. |
| Informed Consent | Written informed consent for publication was obtained from the patient. |
